# Efficacy and safety of finerenone in individuals with type 2 diabetes mellitus complicated by diabetic kidney disease: A retrospective observational study

**DOI:** 10.1016/j.metop.2024.100318

**Published:** 2024-09-07

**Authors:** Yuji Kawaguchi, Yuriko Hajika, Narumi Ashida, Maho Rinka, Chie Hamai, Koji Masumoto, Jun Sawa, Kenji Hamazaki, Yasuro Kumeda

**Affiliations:** Department of Internal Medicine, Minami Osaka Hospital, Osaka, Japan

**Keywords:** Type 2 diabetes mellitus, Diabetic kidney disease, Finerenone, Urinary albumin-to-creatinine ratio, Estimated glomerular filtration rate

## Abstract

**Aim/introduction:**

Early therapeutic interventions are necessary to reduce cardiovascular and renal composite endpoints in individuals with type 2 diabetes mellitus (T2DM) and diabetic kidney disease (DKD). Clinical trials have shown that finerenone suppresses cardiovascular and renal composite endpoints by reducing the urinary albumin-to-creatinine ratio (UACR) and suppressing the decline in the Estimated Glomerular Filtration Rate (eGFR). However, the efficacy and safety of finerenone in real-world clinical practice remain unclear. This study aimed to evaluate the reduction in the UACR as an efficacy endpoint as well as changes in eGFR and serum potassium levels as safety endpoints before and after finerenone administration.

**Materials and methods:**

This retrospective observational study collected data from outpatients with T2DM and DKD upon initiation of finerenone treatment and 3 months after treatment. The primary efficacy endpoint was the change in the UACR from the start of finerenone treatment to after 3 months, while the primary safety endpoints were the changes in serum potassium levels and eGFR over the same period.

**Results:**

The mean UACR significantly decreased from 668.6 mg/gCr at the start of finerenone treatment to 367.8 mg/gCr after 3 months (p < 0.001). Contrastingly, serum potassium levels, eGFRs, systolic and diastolic blood pressures, body mass indices, and HbA1c levels showed no significant changes between treatment initiation and 3 months post-treatment (all p > 0.05).

**Conclusions:**

In individuals with T2DM and DKD, finerenone treatment significantly reduced the UACR, with no post-treatment changes in potassium levels or eGFRs.

**Trial registration:**

This trial was registered with the University Hospital Medical Information Network Clinical Trial Registry (UMIN000054821).

## Highlights

• Finerenone administration significantly reduced the UACR in terms of efficacy.
• Finerenone administration did not increase in potassium levels or eGFR.
• Finerenone may reduce cardiovascular and renal composite endpoints.

## 1 Introduction

Long-term type 2 diabetes mellitus (T2DM) increases the mortality risk due to chronic complications, including macrovascular complications such as ischemic heart disease, cerebrovascular disease, and peripheral arterial disease, as well as microvascular complications such as diabetic kidney disease (DKD), diabetic neuropathy, and diabetic retinopathy [1,2]. Individuals with DKD have a significantly higher risk of cardiovascular events compared to those with chronic kidney disease (CKD) without T2DM [3]. Among individuals with T2DM, those with overt albuminuria and an estimated glomerular filtration rate (eGFR) < 60 mL/min/1.73 m^2^ have a 3.2-fold higher risk of cardiovascular events (including cardiovascular death, nonfatal myocardial infarction, and nonfatal stroke) as well as a 22.2-fold higher risk of renal events (including death due to renal disease, need for dialysis or transplantation, and doubling of serum creatinine levels) compared to those with normoalbuminuria and an eGFR ≥90 mL/min/1.73 m^2^ [4]. Furthermore, the rate of decline in the eGFR is positively correlated with the incidence of end-stage renal disease [5]. Accordingly, multifaceted treatments that manage blood pressure and lipid levels and offer glycemic control, are crucial for preventing renal function decline [6].

Obesity, hypertension, dyslipidemia, and diabetes mellitus activate the renin-angiotensin-aldosterone system, with aldosterone at its downstream end inducing excessive activation of mineralocorticoid receptors [7,8]. CKD progression factors include inadequate glycemic control, elevated intraglomerular pressure, and renal inflammation and fibrosis [9,10]. Mineralocorticoid receptor activation is known to be involved in renal inflammation and fibrosis [10]. For individuals with DKD, sodium-glucose cotransporter 2 inhibitors (SGLT2-i), glucagon-like peptide-1 receptor agonists, or non-steroidal mineralocorticoid receptor antagonists (MRAs) are recommended for reducing the cardiovascular and renal risk [11]. In Japanese individuals with T2DM, hypertension, and microalbuminuria, the administration of esaxerenone in combination with an angiotensin-converting enzyme inhibitor (ACE-i) or angiotensin II receptor blocker (ARB) has been shown to increase the rate of reduction or remission of albuminuria [12].

Finerenone is thought to suppress fibrosis and exert anti-inflammatory effects by binding to mineralocorticoid receptors and inhibiting their excessive activation, thus potentially reducing cardiovascular and renal dysfunction [13]. According to the ARTS-DN trial, which included 823 individuals with T2DM, the urine albumin-to-creatinine ratio (UACR) 90 days after finerenone administration was significantly decreased in the ≥7.5 mg finerenone group compared to the placebo group; further, there was a dose-dependent reduction in albuminuria [14]. In the FIDELIO-DKD trial, which investigated the renal protective effect of finerenone, finerenone treatment of individuals with T2DM and microalbuminuria or macroalbuminuria resulted in an 18 % reduction of the risk of the composite renal endpoint of renal failure, a ≥40 % reduction in eGFR, or death due to renal disease compared with placebo [15]. The FIGARO-DKD trial, which included individuals with earlier DKD than those in the FIDELIO-DKD trial and employed targeted cardiovascular events as the primary endpoint, found that finerenone administration yielded a 13 % reduction in the risk of composite cardiovascular events (cardiovascular death, myocardial infarction, stroke, and hospitalization for heart failure) compared to placebo [16]. Individuals with a >30 % decrease in UACR over 2 years showed a reduced risk of progression to end-stage renal failure compared to those without changes [17]; accordingly, reducing the UACR by >30 % is recommended to slow disease progression among individuals with DKD who have a UACR ≥300 mg/gCr [11]. Furthermore, individuals with DKD who have a UACR of 30–150 mg/gCr have an ≈8.5 times higher risk of progressing to macroalbuminuria over an 8-year observation period compared to those with normoalbuminuria [18]. Therefore, early diagnosis and treatment of DKD are necessary.

However, a previous study using The Real-World Data database showed that among individuals with eGFRs <90 mL/min/1.73 m^2^, only 6.9 % underwent quantitative proteinuria testing, with the diagnosis rate of CKD being 5.9 % among those who did not undergo this testing. This indicates a very low rate of proteinuria evaluation and CKD diagnosis among suspected cases [19]. Therefore, it is important to perform quantitative proteinuria testing and UACR assessments, rather than relying solely on qualitative urine tests, in order to effectively manage kidney disease progression. However, an analysis using a comprehensive database of nearly all electronic prescriptions in Japan revealed that only 67.3 % of individuals underwent qualitative urine testing, while only 19.4 % underwent quantitative proteinuria or UACR testing. This indicates a significant underutilization of these critical diagnostic methods [20]. Although clinical trials such as the ARTS-DN, FIDELIO-DKD, and FIGARO-DKD trials have demonstrated significant reductions in UACRs following finerenone, UACR evaluation is often insufficiently conducted in real-world clinical practice.

It is important to closely monitor for hyperkalemia when administering MRAs such as finerenone, especially in individuals with advanced CKD or those undergoing treatment with ACE-is or ARBs [15]. Additionally, finerenone is considered to reduce intraglomerular pressure by constricting the afferent arterioles and dilating the efferent arterioles, similar to the mechanism of action of SGLT2-i [21], and thus involves the risk of a decrease in eGFR (−3.18 mL/min/1.73 m^2^ at 4 months after administration) [15]. Therefore, it is necessary to carefully monitor renal function during the initial treatment period.

Although the use of MRAs for DKD is recommended, there have been no reports confirming the efficacy and safety of finerenone in Japanese individuals with DKD. Therefore, we aimed to conduct a retrospective observational study of changes from baseline in UACRs, serum potassium levels, and eGFRs in individuals with DKD who received finerenone at Minami Osaka Hospital.

## 2 Materials and methods

### 2.1 The registered participants

This retrospective observational study included individuals with T2DM and DKD who began treatment with finerenone at doses of 10 mg or 20 mg between April 2023 and April 2024 at Minami Osaka Hospital. The inclusion criteria included a diagnosis of T2DM and DKD based on the American Diabetes Association Criteria [11,22] (based on the presence of UACRs ≥30 mg/gCr or reduced eGFR <60 mL/min/1.73 m^2^, or both, in the absence of signs or symptoms of other primary causes of kidney damage) at least 1 year before trial enrollment and eligibility for finerenone treatment. The exclusion criteria included <90 % adherence to finerenone administration and opting out of study enrollment. The observation period was from April 2023 to July 2024, and we collected and analyzed data from 30 individuals who continuously received finerenone. The study protocol was reviewed and approved by the Ethics Committee of Minami Osaka Hospital (approved on August 1, 2024, approval number 2024-5). This trial was registered in the University Hospital Medical Information Network Clinical Trial Registry (UMIN000054821). Participants' consent was obtained by displaying an overview of the study on the Minami Osaka Hospital website and giving patients an opt-out option, with no one refusing participation. This study was conducted in accordance with the Declaration of Helsinki (1975, as revised in 2013).

### 2.2 Outcome measures

The primary efficacy endpoint was the change in UACR from the start of finerenone administration to 3 months after treatment initiation. The primary safety endpoints were the changes in serum potassium levels and eGFRs. Serum potassium levels were measured using the ion-selective electrode method. Further, eGFRs were calculated based on the measured creatinine levels using the enzymatic method, following the Japanese equation for estimating eGFRs [23]. The secondary endpoints were the changes in systolic and diastolic blood pressures, body mass indices (BMIs), and HbA1c levels from the start of finerenone administration to 3 months after treatment initiation, as well as comparison of the decline rate of the UACR and decline in the eGFR based on baseline proteinuria categories (microalbuminuria 30–299 mg/gCr; A2, macroalbuminuria ≥300 mg/gCr; A3) and baseline eGFR categories (eGFR ≥60; G1-G2, eGFR< 60; G3). The efficacy and safety of the primary and secondary endpoints were also analyzed in subgroups of individuals based on baseline median age (<70 and ≥ 70 years), sex, use or non-use of renin-angiotensin system inhibitors (RAS-is), and use or non-use of SGLT2-i.

### 2.3 Statistical analysis

Data are presented as means ± standard deviations unless otherwise noted. Comparisons of UACRs, serum potassium levels, eGFRs, blood pressures, BMIs, and HbA1c levels before and after finerenone treatment were conducted using paired t-tests. Comparisons of the reduction in UACRs based on the baseline proteinuria and eGFR categories were conducted using Student's t-tests. Statistical analyses were performed using EZR v. 1.37 (Saitama Medical Center, Jichi Medical University, Saitama, Japan) [24]. Statistical significance was set at p < 0.05.

## 3 Results

### 3.1 Participant characteristics

Table 1 shows the characteristics of the study participants. We included 30 individuals with T2DM and DKD, excluding one individual whose adherence rate for finerenone was <90 %. The mean age of the participants was 67.2 years. The cohort included 24 males and 6 females. The mean disease duration of T2DM was 9.7 years. The comorbidities included hypertension in 27 (90.0 %) participants and dyslipidemia in 24 (80.0 %) participants. Regarding the baseline parameters, the mean BMI was 26.0 kg/m^2^, the mean HbA1c level was 7.1 %, eGFR was 64.5 mL/min/1.73 m^2^ (eGFR ≥60 mL/min/1.73 m^2^; 16 [53.3 %] individuals in the G1-G2 group and eGFR <60 mL/min/1.73 m^2^; 14 [46.7 %] individuals in the G3 group), the mean UACR was 668.6 mg/gCr (microalbuminuria of 30–299 mg/gCr; 10 [33.3 %] individuals in the A2 group and macroalbuminuria of ≥300 mg/gCr; 20 [66.7 %] individuals in the A3 group), and the mean serum potassium level was 4.1 mEq/L. The finerenone starting dose was 20 mg and 10 mg for individuals with eGFRs ≥60 mL/min/1.73 m^2^ and <60 mL/min/1.73 m^2^, respectively. All participants whose finerenone starting dose was 10 mg had serum potassium levels ≤4.8 mEq/L and a <30 % decrease in eGFR after 1 month of treatment; therefore, their dose was increased to 20 mg. Finerenone is generally administered to individuals receiving ACE-is or ARBs, except in cases where treatment with these drugs is not appropriate. However, in our study, 14 (46.7 %) individuals were receiving ARBs, and 9 (33.3 %) individuals were receiving Angiotensin Receptor Neprilysin Inhibitors (ARNIs). This can be attributed to the fact that 27 (90.0 %) individuals had comorbid hypertension, and thus, RAS-is or ARNIs could not be used given the risk of excessive lowering of blood pressure. No individuals made changes to their diabetes mellitus or hypertension treatment during the observation period.

**Table 1:** Baseline characteristics of participants (n = 30).

baseline characteristics	Participant data
Age (y)	67.2 ± 11.0
Sex, male, n (%)	24 (80.0)
Duration of diabetes mellitus (y)	9.7 ± 5.7
Hypertension, n (%)	27 (90.0)
Dyslipidemia, n (%)	24 (80.0)
BMI (kg/m^2^)	26.0 ± 3.4
HbA1c (%)	7.1 ± 1.0
Abumin (g/dL)	4.2 ± 0.4
eGFR (mL/min/1.73m^2^)	64.5 ± 22.6
eGFR ≥60; G1-G2, n (%)	16 (53.3)
eGFR <60; G3, n (%)	14 (46.7)
UACR (mg/gCr)	668.6 ± 592.4
UACR (30–299 mg/gCr), n (%)	10 (33.3)
UACR (300 mg/gCr <), n (%)	20 (66.7)
Potassium (mEq/L)	4.1 ± 0.5
HDL-C (mg/dL)	45.1 ± 14.8
Triglyceride (mg/dL)	122.2 ± 43.8
LDL-C (mg/dL)	101.6 ± 31.2
Systolic blood pressure (mmHg)	126.9 ± 9.5
Diastolic blood pressure (mmHg)	71.5 ± 13.0
Antihypertensive drugs	
Calcium channel blockers, n (%)	22 (73.3)
Angiotensin receptor blockers, n (%)	14 (46.7)
Angiotensin receptor neprilysin inhibitor, n (%)	9 (33.3)
Beta blockers, n (%)	2 (6.7)
Loop diuretics, n (%)	1 (3.3)
Dyslipidemia treatment medications	
Statins, n (%)	22 (73.3)
Ezetimibe, n (%)	4 (13.3)
Pemafibrate, n (%)	9 (30.0)
Antihyperglycemic drugs	
Sulfonylurea, n (%)	10 (33.3)
Metformin, n (%)	17 (56.7)
SGLT2-i, n (%)	23 (76.7)
DPP-4 inhibitor, n (%)	16 (53.3)
GLP-1RA, n (%)	9 (30.0)
Imeglimin, n(%)	6 (20.0)
Insulin, n (%)	7 (23.3)

BMI, body mass index; HbA1c, glycated hemoglobin; eGFR, estimated glomerular filtration rate; UACR, urinary albumin-to-creatinine ratio; HDL-C, high-density lipoprotein cholesterol; LDL-C, low-density lipoprotein cholesterol; SGLT2-i, sodium-glucose cotransporter 2 inhibitor; DPP-4, dipeptidyl peptidase-4; GLP-1RA, glucagon-like peptide-1 receptor agonists. Data are presented as mean ± standard deviation or number (%).

### 3.2 Evaluation of the efficacy and safety of finerenone

Table 2 shows the comparison of various parameters between the start of finerenone administration and 3 months after treatment initiation. The mean UACR significantly decreased from 668.6 mg/gCr at the baseline to 367.8 mg/gCr after 3 months (p < 0.001), while the mean serum potassium levels, eGFRs, systolic and diastolic blood pressures, BMIs, and HbA1c levels showed no significant post-treatment changes (all p > 0.05). These results were similar based on the baseline median age (<70 and ≥ 70 years), sex, use or non-use of RAS-is, and use or non-use of SGLT2-i (Table S1). However, due to the small number of female patients and non-use of RAS-is, no significant changes in UACRs were observed.

**Table 2:** Changes in UACR, potassium, eGFR, blood pressure, BMI, and HbA1c before and 3 months after finerenone treatment.

	Pre-treatment	Post-treatment	p-value
UACR (mg/gCr)	668.6 ± 592.4	367.8 ± 362.5	<0.001^a^
Potassium (mEq/L)	4.1 ± 0.5	4.2 ± 0.5	0.594
eGFR (mL/min/1.73m^2^)	64.5 ± 22.6	64.2 ± 23.1	0.863
Systolic blood pressure (mmHg)	126.9 ± 9.5	127.5 ± 9.4	0.663
Diastolic blood pressure (mmHg)	71.5 ± 13.0	68.6 ± 13.0	0.187
BMI (kg/m^2^)	26.0 ± 3.4	26.0 ± 3.5	0.979
HbA1c (%)	7.1 ± 1.0	7.0 ± 0.8	0.349

UACR, urinary albumin-to-creatinine ratio; eGFR, estimated glomerular filtration rate; BMI, body mass index; HbA1c, glycated hemoglobin.

Data are presented as the mean ± SD. Pre- and post-treatment measurements are compared by paired t-tests. A p-value of <0.05 was considered significant.

a indicates a statistically significant difference between time points.

Table 3 shows the comparison of the decline rate in UACRs and the decline in eGFRs according to proteinuria and eGFR categories. There were no significant differences in the decline rate of UACRs and the decline in eGFRs (p > 0.05).

**Table 3:** Decline rate of UACR and decline in eGFR according to proteinuria and eGFR category.

Albuminuria category	Microalbuminuria group (30–299 mg/gCr; A2) (n = 10)	Macroalbuminuria group (>300 mg/gCr; A3) (n = 20)	p-value
Decline rate of UACR (%)	46.9 ± 11.9	46.8 ± 12.8	0.989
Decline in eGFR (mL/min/1.73m^2^)	2.3 ± 15.9	−1.6 ± 6.0	0.338
eGFR category	eGFR ≥60; G1-G2 (n = 16)	eGFR <60; G3 (n = 14)	
Decline rate of UACR (%)	48.4 ± 12.6	45.1 ± 12.2	0.472
Decline in eGFR (mL/min/1.73m^2^)	−3.1 ± 9.4	2.9 ± 10.6	0.111

UACR, urinary albumin-to-creatinine ratio; eGFR, estimated glomerular filtration rate. Data are presented as the mean ± SD. Data between groups are compared by student's t-test. A p-value of <0.05 was considered significant.

No other adverse events, such as hypotension, hyponatremia, or hyperuricemia, were observed during the observation period.

## 4 Discussion

This is the first study to evaluate the efficacy of finerenone with respect to improving UACRs as well as its safety with respect to changes in serum potassium levels and eGFRs among Japanese individuals with T2DM and DKD in real-world clinical practice. We observed a significant decrease in UACRs at 3 months post-treatment. In the FIDELIO-DKD trial [15], which mainly targeted patients with CKD stages 3–4 who had overt albuminuria, finerenone resulted in an 18 % risk reduction of the primary renal composite endpoint (renal failure, sustained eGFR decline of ≥40 % from baseline for ≥4 weeks, and renal death) compared to placebo (p = 0.0014). Regarding the exploratory endpoint, finerenone yielded a significant 31 % reduction in UACR from baseline to 4 months post-treatment compared to placebo, which was maintained with finerenone beyond the 4-month period. In the FIGARO-DKD trial [16], which targeted patients with relatively early CKD, approximately half of the individuals had CKD stages 1–2 or had microalbuminuria. There was no significant difference in terms of the secondary renal composite endpoint. However, finerenone tended to have a lower rate than placebo, with a hazard ratio of 0.87 (95 % confidence interval 0.76–1.01) (p = 0.0689). In the FIGARO-DKD trial, the change in UACR from baseline to 4 months post-treatment was significantly reduced by 32 % in the finerenone group compared to the placebo group. Therefore, finerenone is expected to reduce the UACR regardless of the CKD stage. In our study, which included individuals with eGFR categories G1–G3, the UACR significantly decreased by 45.0 % from 668.6 mg/gCr at baseline to 367.8 mg/gCr after 3 months. The Standards of Care in Diabetes 2024 recommend reducing the UACR by ≥ 30 % in individuals with DKD and UACR ≥300 mg/gCr in order to slow CKD progression [11]. Post-hoc analyses of the FIDELIO-DKD and FIGARO-DKD trials revealed that a >30 % reduction in the UACR mediated 84 % and 37 % of the treatment effect on kidney and cardiovascular outcomes, respectively [25]. Therefore, the 45 % reduction in the UACR observed after 3 months of finerenone treatment in our study may have a favorable impact on the future progression of cardiovascular and renal disease. The significant reduction in the UACR observed in our study could be attributed to the fact that all our participants received 20 mg of finerenone, while the finerenone dose in the FIDELIO-DKD and FIGARO-DKD trials was 10–20 mg. Administration of finerenone (1.25 mg, 2.5 mg, 5 mg, 7.5 mg, 10 mg, 15 mg, or 25 mg) or placebo for 90 days demonstrated that finerenone reduces the UACR in a dose-dependent manner [14]. Accordingly, unless there is hyperkalemia or an extremely reduced eGFR, it would be useful to maximally increase the finerenone dose for each individual in order to reduce cardiac and renal endpoints.

Regarding safety, we observed no significant difference in the mean serum potassium levels before and after finerenone administration (4.1 mEq/mL and 4.2 mEq/mL, respectively) (p > 0.05). In the FIDELIO-DKD trial [15], the mean change in serum potassium levels from baseline to 4 months post-treatment was +0.25 mEq/mL. Multivariate analysis of risk factors for serum potassium levels >5.5 mEq/L revealed that the hazard ratios were 1.53 for serum potassium levels >4.5 to ≤4.8 mEq/mL, 2.78 for serum potassium levels >4.8 to ≤5.0 mEq/mL, 4.18 for serum potassium levels >5.0 mEq/mL, 1.51 for eGFRs ≥25 to <45 mL/min/1.73 m^2^, 2.04 for eGFRs <25 mL/min/1.73 m^2^ at the start of finerenone administration, and 1.12 for those with a doubling of UACR. At the start of finerenone administration, it is necessary to closely monitor the serum potassium level, eGFR, and UACR, as well as perform regular follow-ups.

In our study, we observed no significant difference in the mean eGFR before and after finerenone administration (64.5 mL/min/1.73 m^2^ and 64.2 mL/min/1.73 m^2^, respectively) (p > 0.05). Finerenone is considered to reduce the intraglomerular pressure by constricting and dilating the afferent and efferent arterioles, respectively, similar to the effect of SGLT2-i [21]. Although attention should be paid to the initial eGFR dip, the slope of the decline in the eGFR with long-term administration is gentler than with placebo. In the FIDELIO-DKD trial [15], the decline in the eGFR over the first 4 months was −0.73 (−1.03 to −0.44) mL/min/1.73 m^2^ for placebo and −3.18 (−3.44 to −2.91) mL/min/1.73 m^2^ for finerenone. However, after 4 months, the annual decline in the eGFR was −3.97 (−4.27 to −3.66) mL/min/1.73 m^2^ for placebo and −2.66 (−2.96 to −2.36) mL/min/1.73 m^2^ for finerenone. Beyond 24 months of treatment, the decline in the eGFR with finerenone reversed compared to placebo, which contributed to differences in renal composite endpoints.

In this study, we observed no significant difference in systolic blood pressure before and after finerenone administration. In a report using the FIDELIO-DKD trial dataset [26], in the overall population, treatment with finerenone significantly reduced blood pressure by −3.84 mmHg compared with placebo after 4 months. However, when the baseline systolic blood pressure was grouped into quartiles (Q) (Q1, ≤128.7 mmHg; Q2, >128.7 to ≤138.3 mmHg; Q3, >138.3 to ≤148.0 mmHg; Q4, >148.0 mmHg), the changes in systolic blood pressure from baseline to 4 months after finerenone administration were +4.99, −0.85, −5.63, and −11.76 mmHg, respectively. This suggests that the baseline systolic blood pressure is positively correlated with the antihypertensive effect. In the present study, the baseline systolic blood pressure was 126.9 mmHg, suggesting that blood pressure was well-managed. This may explain the lack of an observed antihypertensive effect with finerenone administration.

The above results were observed in subgroup analyses of this study, regardless of age, sex, or the concomitant use of RAS-is or SGLT2-i. The FIDELIO-DKD trial [15] and FIGARO-DKD trial [16] were initiated before the efficacy of SGLT2-i for DKD was established, and concomitant use of an SGLT2-i was low (6.7 %). The decline rate of UACRs was nearly the same between the use and non-use of SGLT2-i. On the other hand, this study examined the efficacy and safety of adding finerenone to treatment in accordance with the latest guidelines, and the baseline rate of SGLT2-i use was 76.7 %. This similar result suggests that the efficacy and safety of combining finerenone with SGLT2-i in clinical practice can be evaluated. Furthermore, in the FIDELIO-DKD trial [15] and the FIGARO-DKD trial [16], the inclusion criteria required the administration of an RAS-i at the maximum tolerated dose. In contrast, in this study, an RAS-i was not administered in individuals in whom the attending physician determined it was unnecessary, resulting in a baseline usage rate of 80 %. This study reflects real-world clinical practice and demonstrates that finerenone can be used safely regardless of RAS-i use or non-use.

In the present study, the decline rate of UACRs and the decline in eGFRs did not significantly differ according to the baseline proteinuria and eGFR categories. In the FIGARO-DKD study, subgroup analysis of the UACR at 4 months after finerenone administration revealed a similar reduction in the UACR, with 33 % and 39 % in the microalbuminuria and macroalbuminuria groups, respectively [27]. The United Kingdom Prospective Diabetes Study (UKPDS 64) reported that the risk of death from cardiovascular disease increased with progression of nephropathy, with annual mortality rates of 2.0 % in individuals with microalbuminuria, 3.5 % in individuals with macroalbuminuria, and 12.1 % in individuals with elevated plasma creatinine or receiving renal replacement therapy [28]. This demonstrates the importance of regular quantification of albuminuria in individuals with T2DM; moreover, appropriate therapeutic interventions should be initiated at the early stage of nephropathy when microalbuminuria is present. However, in individuals with overt albuminuria, glomerular inflammation is induced, and excessive activation of mineralocorticoid receptors and aldosterone upregulation may contribute to kidney damage [7]. Given that finerenone may reduce glomerular inflammation and fibrosis by blocking mineralocorticoid receptors [29], finerenone may also be effective for individuals with advanced nephropathy. As mentioned above, attention should be paid to the initial eGFR dip when finerenone is first administered, but since no significant decrease was observed regardless of albuminuria or eGFR categories, finerenone can be administered safely. However, it should be noted that regular measurement of eGFR is necessary to avoid adverse events. In this study, we demonstrated that finerenone administration in a real-world clinical setting for individuals with DKD led to a significant reduction in the UACR, similar to that observed in international phase III trials such as the FIDELIO-DKD trial [15] and the FIGARO-DKD trial [16] while confirming safety by showing no significant changes in serum potassium levels or eGFRs.

This study had some limitations. First, this was a single-center study with a small sample size (n = 30). Second, this was a retrospective observational study with a short observational period of 3 months. Third, there was no comparison between the finerenone-treated and non-treated groups. Comparing finerenone-treated and non-treated groups in individuals with DKD in retrospective observational studies can be difficult to match for background factors and may result in confounding bias. Therefore, to clarify the combined cardiovascular and renal endpoints of finerenone in clinical practice, it is necessary to conduct prospective, double-arm, longer-term, and multicenter collaborative studies with a larger sample size.

## 5 Conclusions

In individuals with T2DM and DKD, finerenone administration significantly reduced the UACR in terms of efficacy, with no observed increase in potassium levels or eGFRs in terms of safety. Our findings suggest that the reduction in cardiovascular and renal composite endpoints observed in large-scale clinical trials of finerenone may also be achievable in real-world clinical practice.

## Funding

This research did not receive any specific grant from funding agencies in the public, commercial, or not-for-profit sectors.

## CRediT authorship contribution statement

**Yuji Kawaguchi:** Writing – original draft, Methodology, Conceptualization. **Yuriko Hajika:** Writing – original draft, Formal analysis. **Narumi Ashida:** Visualization. **Maho Rinka:** Validation, Resources. **Chie Hamai:** Resources. **Koji Masumoto:** Resources. **Jun Sawa:** Visualization. **Kenji Hamazaki:** Resources, Data curation. **Yasuro Kumeda:** Writing – review & editing, Methodology, Conceptualization.

## Declaration of competing interest

The authors declare that they have no known competing financial interests or personal relationships that could have appeared to influence the work reported in this paper.
