# Supplementary material for: Efficacy and safety of finerenone in individuals with type 2 diabetes mellitus complicated by diabetic kidney disease: A retrospective observational study
Source: Metabol Open. 2024 Sep 7;24:100318. doi: 10.1016/j.metop.2024.100318 (PMC11417194; doi:10.1016/j.metop.2024.100318)
Supplement: Multimedia component 1 [file mmc1.docx]

**Table S1**. Changes in UACR, potassium, eGFR, blood pressure, BMI, and HbA1c before and 3 months after finerenone treatment. Subgroup analysis based on baseline median age (< 70 and ≥ 70 years), sex, use or non-use of RAS-i, and use or non-use of SGLT2-i

|  | Pre-treatment | Post-treatment | p-value |
| --- | --- | --- | --- |
| **Age <70 years (n=15)** |  |  |  |
| UACR (mg/gCr) | 595.9 ± 583.6 | 323.6 ± 363.0 | 0.001* |
| Potassium (mEq/L) | 4.2 ± 0.5 | 4.2 ± 0.5 | 0.276 |
| eGFR (mL/min/1.73m^2^) | 69.2 ± 24.2 | 70.4 ± 24.3 | 0.606 |
| Systolic blood pressure (mmHg) | 128.9 ± 7.6 | 129.9 ± 7.5 | 0.645 |
| Diastolic blood pressure (mmHg) | 75.7 ± 9.1 | 73.7 ± 11.2 | 0.338 |
| BMI (kg/m^2^) | 27.2 ± 4.0 | 27.3 ± 4.2 | 0.766 |
| HbA1c (%) | 6.8 ± 0.8 | 7.0 ± 0.9 | 0.284 |
| **Age ≥ 70 years (n=15)** |  |  |  |
| UACR (mg/gCr) | 741.2 ± 603.7 | 412.1 ± 372.2 | <0.001* |
| Potassium (mEq/L) | 4.1 ± 0.4 | 4.1 ± 0.4 | 0.899 |
| eGFR (mL/min/1.73m^2^) | 59.8 ± 20.6 | 57.9 ± 18.5 | 0.537 |
| Systolic blood pressure (mmHg) | 124.8 ± 11.7 | 125.1 ± 11.7 | 0.896 |
| Diastolic blood pressure (mmHg) | 67.3 ± 13.4 | 63.5 ± 11.6 | 0.345 |
| BMI (kg/m^2^) | 24.7 ± 2.9 | 24.7 ± 2.9 | 0.699 |
| HbA1c (%) | 7.4 ± 1.4 | 7.0 ± 0.9 | 0.127 |
| **Male (n=24)** |  |  |  |
| UACR (mg/gCr) | 638.3 ± 535.3 | 334.1 ± 321.1 | <0.001* |
| Potassium (mEq/L) | 4.1 ± 0.5 | 4.2 ± 0.4 | 0.347 |
| eGFR (mL/min/1.73m^2^) | 64.7 ± 22.7 | 64.0 ± 23.2 | 0.730 |
| Systolic blood pressure (mmHg) | 127.0 ± 10.2 | 126.6 ± 10.6 | 0.838 |
| Diastolic blood pressure (mmHg) | 71.2 ± 12.8 | 68.0 ± 12.6 | 0.234 |
| BMI (kg/m^2^) | 25.1 ± 3.2 | 25.0 ± 3.1 | 0.465 |
| HbA1c (%) | 7.2 ± 1.2 | 7.1 ± 0.9 | 0.485 |
| **Female (n=6)** |  |  |  |
| UACR (mg/gCr) | 789.0 ± 816.1 | 502.8 ± 516.6 | 0.070 |
| Potassium (mEq/L) | 4.4 ± 0.2 | 4.3 ± 0.4 | 0.649 |
| eGFR (mL/min/1.73m^2^) | 63.6 ± 24.4 | 65.0 ± 19.0 | 0.726 |
| Systolic blood pressure (mmHg) | 126.5 ± 9.6 | 131.0 ± 6.2 | 0.187 |
| Diastolic blood pressure (mmHg) | 72.8 ± 9.0 | 71.2 ± 12.0 | 0.542 |
| BMI (kg/m^2^) | 29.5 ± 3.5 | 29.8 ± 3.9 | 0.402 |
| HbA1c (%) | 6.7 ± 0.9 | 6.6 ± 0.7 | 0.368 |
| **Use of RAS-i (n=24)** |  |  |  |
| UACR (mg/gCr) | 725.9 ± 607.0 | 395.2 ± 379.3 | <0.001* |
| Potassium (mEq/L) | 4.2 ± 0.5 | 4.2 ± 0.4 | 0.809 |
| eGFR (mL/min/1.73m^2^) | 65.4 ± 24.2 | 65.2 ± 23.6 | 0.900 |
| Systolic blood pressure (mmHg) | 128.5 ± 8.8 | 128.3 ± 9.1 | 0.858 |
| Diastolic blood pressure (mmHg) | 73.9 ± 11.9 | 69.8 ± 13.2 | 0.124 |
| BMI (kg/m^2^) | 26.4 ± 3.6 | 26.4 ± 3.8 | 0.810 |
| HbA1c (%) | 7.0 ± 1.0 | 6.9 ± 0.8 | 0.584 |
| **Non-use of RAS-i (n=6)** |  |  |  |
| UACR (mg/gCr) | 439.3 ± 481.8 | 258.6 ± 298.2 | 0.068 |
| Potassium (mEq/L) | 4.0 ± 0.5 | 4.1 ± 0.5 | 0.552 |
| eGFR (mL/min/1.73m^2^) | 60.8 ± 16.1 | 60.0 ± 16.1 | 0.917 |
| Systolic blood pressure (mmHg) | 120.2 ± 12.2 | 124.5 ± 13.4 | 0.202 |
| Diastolic blood pressure (mmHg) | 61.8 ± 7.1 | 63.8 ± 6.7 | 0.381 |
| BMI (kg/m^2^) | 24.3 ± 3.6 | 24.4 ± 3.5 | 0.641 |
| HbA1c (%) | 7.6 ± 1.7 | 7.3 ± 1.2 | 0.278 |
| **Use of SGLT2-i (n=23)** |  |  |  |
| UACR (mg/gCr) | 715.7 ± 635.0 | 390.4 ± 392.3 | <0.001* |
| Potassium (mEq/L) | 4.1± 0.5 | 4.2 ± 0.4 | 0.418 |
| eGFR (mL/min/1.73m^2^) | 66.5 ± 23.4 | 66.2 ± 22.9 | 0.894 |
| Systolic blood pressure (mmHg) | 126.3 ± 10.9 | 126.7 ± 10.4 | 0.833 |
| Diastolic blood pressure (mmHg) | 73.4 ± 11.7 | 70.0 ± 12.7 | 0.220 |
| BMI (kg/m^2^) | 26.2 ± 4.1 | 26.2 ± 4.2 | 0.817 |
| HbA1c (%) | 7.1 ± 1.3 | 6.9 ± 0.8 | 0.293 |
| **Non-use of SGLT2-i (n=7)** |  |  |  |
| UACR (mg/gCr) | 513.8 ± 395.8 | 293.9 ± 260.4 | 0.007* |
| Potassium (mEq/L) | 4.2 ± 0.4 | 4.1 ± 0.4 | 0.779 |
| eGFR (mL/min/1.73m^2^) | 57.9 ± 20.2 | 57.6 ± 19.3 | 0.852 |
| Systolic blood pressure (mmHg) | 128.9 ± 6.0 | 130.3 ± 8.4 | 0.361 |
| Diastolic blood pressure (mmHg) | 65.1 ± 11.6 | 64.1 ± 10.7 | 0.593 |
| BMI (kg/m^2^) | 25.0 ± 1.6 | 25.1 ± 1.8 | 0.643 |
| HbA1c (%) | 7.1 ± 0.9 | 7.2 ± 1.0 | 0.906 |

UACR, urinary albumin-to-creatinine ratio; eGFR, estimated glomerular filtration rate; BMI, body mass index; HbA1c, glycated hemoglobin, RAS-i; renin-angiotensin system inhibitor, SGLT2-i; sodium-glucose cotransporter 2 inhibitor.

Data are presented as the mean ± SD. Pre- and post-treatment measurements are compared by paired t-tests. A p-value of < 0.05 was considered significant.

*Indicates a statistically significant difference between time points.
